# Anticoagulants and the Hemostatic System: A Primer for Occupational Stress Researchers

**DOI:** 10.3390/ijerph182010626

**Published:** 2021-10-11

**Authors:** Eamonn Arble, Bengt B. Arnetz

**Affiliations:** 1 Department of Psychology, Eastern Michigan University, Ypsilanti, MI 48197, USA; 2 Department of Family Medicine, College of Human Medicine, Michigan State University, Grand Rapids, MI 49503, USA; arnetzbe@msu.edu

**Keywords:** anticoagulants, hemostasis, stress, occupational stress

## Abstract

Anticoagulation, the body’s mechanism to prevent blood clotting, is an internal biomarker of an individual’s response to stress. Research has indicated that understanding the causes, processes, and consequences of anticoagulation can provide important insight into the experience of individuals facing emotional and occupational strain. Unfortunately, despite their importance, the mechanisms and implications of anticoagulation are unfamiliar to many researchers and practitioners working with trauma-exposed professionals. This paper provides an accessible primer on the topic of anticoagulation, including an overview of the biological process, the research connecting these processes with emotional and occupational functioning, as well as some potential methods for assessment.

## 1. Introduction

The biological responses to stress are myriad and complex. This complexity is amplified by the consideration of psychological processes, which can affect the experience, response, and impact of physiological reactions. For researchers, these integrated biopsychosocial mechanisms provide countless opportunities for clever means of assessment. By capturing underlying biological data, richer insight into cognitive and emotional functioning can be obtained, while overcoming some of the limitations of more traditional self-reports. Unfortunately, this complexity can also serve as a barrier to practitioners and researchers, as surveying vast and widely discrepant fields of knowledge can prove near impossible. As a result, useful biomarkers may be relatively neglected within certain domains of research.

We wish to highlight one such biological process: the link between anticoagulation and stress or trauma. Circulating levels of anticoagulants may provide a useful indicator of stress reactivity and response, supplementing other more common measures such as cortisol and heart rate. In this primer on the topic of anticoagulants, we will provide a brief description of the relevant biological processes, as well as a summary of the literature indicating its relevance for both personal and occupational health.

## 2. Hemostasis and the Hemostatic System

**Figure 1:**
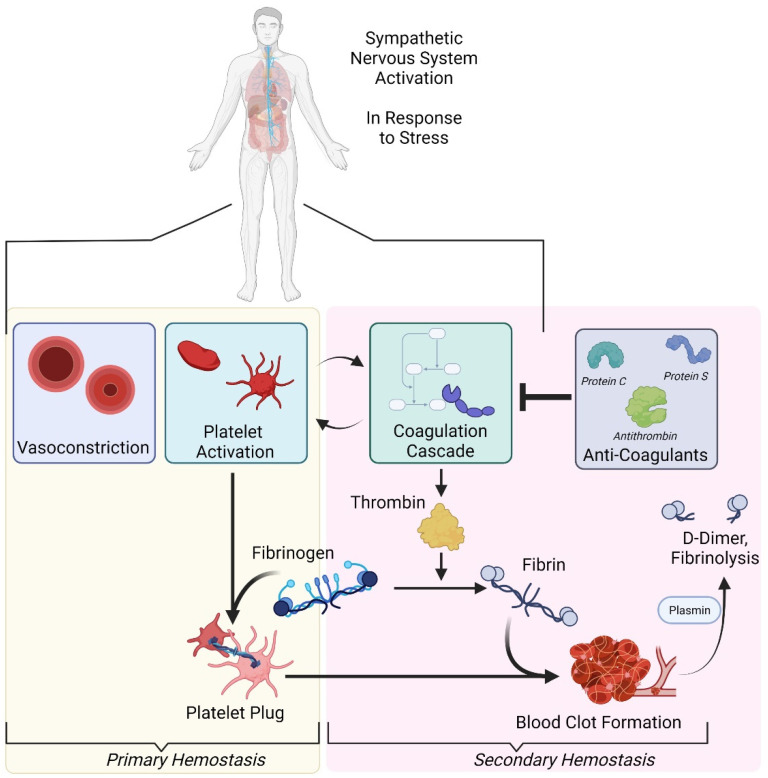
A visual depiction of hemostasis and the hemostatic response. The image was created with BioRender.com (accessed on 27 September 2021).

An understanding of coagulation begins with hemostasis and the hemostatic system. The circulation of blood with finely balanced viscosity is critical for healthy functioning [1]. In the case of injury, hemorrhaging must be limited, and the mechanism to ensure this is known as hemostasis [2]. This normal and adaptive process is adaptive but may also become excessively activated, leading to a maladaptive disruption of blood flow. For example, if the viscosity of the blood is too thick or if there are imperfections in blood vessels, blood clots might be formed. Thus, blood viscosity can be either pathologically uninhibited (i.e., hemorrhaging) or pathologically inhibited (i.e., thrombosis). The system to manage this balance is known as the hemostatic system, consisting of coagulation, fibrinolysis, and platelets. Both hemostasis and the hemostatic system are essential for understanding the role of anticoagulants. For a visual reference of this process, please consult Figure 1.

In the case of injury, blood loss becomes an immediate concern for the body to address, as it can ultimately prove fatal. When a blood vessel is damaged, the blood contained therein will begin to “leak out” (i.e., hemorrhaging, which can be either internal or external). Fortunately, hemostasis, the body’s system of coagulation and platelets, activates in response to injury [3].

In what is referred to as “primary hemostasis”, the response of platelets is key [4]. Platelets normally circulate within the blood, neither adhering to structures nor excessively congregating [5]. In the case of damage to the interior wall of a blood vessel, also known as the endothelium, platelets will begin to adhere to the subendothelial collagen. As platelets begin to “stick” to the site of the injury, they become activated. This activation leads to the release of various products (e.g., serotonin) that are designed to facilitate further platelet aggregation [6]. In effect, these agents attract more platelets to the damaged area that, in turn, adhere to the already attached platelets, become activated, and support a continuous cycle of platelet attraction and activation.

To illustrate this process, one might imagine a leak in a large bucket. Each platelet moving to the injury represents a piece of tape being placed over the leak. One piece of tape is likely to prove insufficient, so additional pieces are required. As the platelets aggregate, more and more pieces of tape are placed over the hole, until there is a sufficient number of pieces to hold back the water. This accumulation is referred to as the platelet plug [7].

While the platelet plug is forming, “secondary hemostasis” can begin [8]. This process is defined by the “coagulation cascade”, in which clotting factors within blood plasma are activated (the chain of activations across these factors, with one activating the next, is why the process is referred to as a cascade) [9]. Down the sequence of these activations, the enzyme thrombin is activated, leading to the “thrombin burst”. Thrombin is now released very rapidly, resulting in several important consequences. Thrombin expression induces further activation of platelets and clotting factors, but perhaps most importantly, will lead to the formation of fibrin.

Fibrin will organize in longitudinal and lateral strands, forming a three-dimensional mesh that stabilizes the platelet plug. The formation of this mesh also incorporates platelets and blood cells within its structure, causing the platelet plug to harden [10]. The plug is subsequently referred to as a “thrombus” or “clot”. Returning to the example of placing tape over a leaking bucket, in secondary hemostasis, a patch is now layered over the tape as a final seal.

The creation of the clot, or thrombus, reflects the efforts of a defensive healing system. Unfortunately, excessive and abnormal formation of blood clots (i.e., thrombosis) carries potentially severe consequences. Over 100 years ago, the physician Rudolf Virchow began a critical exploration of the factors contributing to thrombus formation, with modern research identifying three critical components known as Virchow’s triad: (1) hypercoagulability (i.e., abnormal blood constituents); (2) endothelial injury (i.e., vessel wall abnormalities); and (3) hemodynamic changes (i.e., abnormal blood flow) [11]. These three physiological indicators all place an individual at risk for thrombosis, which can subsequently result in a number of pathologies [12]. For example, if the clot becomes dislodged from the vessel wall and enters the circulatory system, serious medical complications such as stroke and heart attack are possible.

The final step of the hemostatic process, fibrinolysis, is designed to dissolve the clot without allowing its escape into the circulation. Incorporated within the clot is the enzyme plasminogen, which has an affinity for fibrin. When activated, plasminogen is converted to its active form, plasmin [13]. This activation is driven by tissue Plasminogen Activator (tPA) or urokinase Plasminogen Activator (uPA), the former being released by endothelial cells (specifically, the damaged endothelium of a blood vessel). Both tPA and uPA are regulated by the presence of plasminogen activator inhibitors PAI-I and PAI-2, maintaining the balance that is critical to the hemostatic system [14].

As the plasmin is activated, it begins to cut at the fibrin strands that comprise the mesh stabilizing the platelet plug. The plasmin will continue this work until the clot is dissolved, leaving only tiny remaining fragments. These soluble remains are known as fibrin degradation products (FDPs). A notable FDP is D-dimer, whose fragments provide an indication of the degree of thrombosis. Concentrations of D-dimer have become a useful indicator of the functioning of fibrinolytic systems [15].

## 3. The Role of Anticoagulants

The activation of thrombin is a critical step in blood coagulation and subsequent clotting. However, as noted previously, excessive blood coagulation (i.e., excessive inhibition of blood flow) is also harmful. To prevent this excess activity, the body produces a series of natural anticoagulants such as Protein C, Protein S, and antithrombin [16]. These anticoagulants limit the activation of thrombin, thereby preventing thrombin’s ability to transform fibrinogen into fibrin and resulting in a disruption of the mesh stabilizing the platelet plug. Furthermore, substances such as antithrombin inactivate other coagulation factors, again limiting the amount of coagulation taking place [17].

These substances combine with other biological mechanisms that serve an anticoagulant function. For instance, vascular endothelial cells are wrapped in a gel-like structure known as the glycocalyx. The glycocalyx acts as a barrier between these cells and the circulating components of the blood [18]. In addition to its protective function, the glycocalyx regulates vascular permeability and prevents clotting activation [19]. Furthermore, the intact endothelium has several anticoagulant mechanisms, including the secretion of heparan sulfate, which enhances the anticoagulant properties of antithrombin. Unfortunately, this protective barrier is highly susceptible to disruption (and subsequent thinning) from vascular pathologies and stress [20,21].

In summary, anticoagulation plays a key role in preventing blood flow from becoming pathologically inhibited and serves as a marker of healthy recovery.

## 4. An Evolutionary Perspective on Anticoagulants in Response to Trauma

Understanding the important role of anticoagulants in response to injury may initially appear less relevant to researchers or clinicians interested in emotional and psychological processes. After all, many people work with trauma-exposed professionals outside of the context of physical injury. However, these processes are not only relevant in the case of physical injury but are also important for understanding an individual’s response to stress. Specifically, there appears to be significant stress-related changes to the hemostatic system that can impact thrombosis.

The experience of stress may place individuals in a “prothrombotic state”; their bodies may be primed to engage in the wound-healing processes described above. Individuals undergoing stress may demonstrate hypercoagulability, hyperactive platelets, and attenuated fibrinolysis [22]. From an evolutionary perspective, this state of readiness makes sense. If an individual is experiencing stress, say, for example, they are being hunted by a predatory animal, the “fight or flight” mechanism is engaged. In either case (fighting or fleeing), the potential for injury is elevated. If an individual is running or fighting and sustains an injury, excessive bleeding places them in grave danger. Thus, the body’s adoption of a prothrombotic state is meant to provide a readiness to address this potentially lethal contingency [23]. Moreover, since the system is designed to manage injuries succumbed in a state of fight or flight, the body will be physically active, involved in fighting or fleeing, which counteracts the risk for blot clots due to the increased thrombolytic activity.

## 5. Anticoagulant Links to Stress

The prothrombotic readiness that accompanies sympathetic nervous system activation can prove detrimental if it is excessive (as is the case for essentially all biological processes). However, it is perhaps most detrimental when it is removed from the fight or flight context. An individual who needs to fight to survive may indeed derive utility from a readiness to prevent bleeding, but what is the benefit for an individual maintaining this readiness in the face of daily occupational stressors? Entering a state of hypercoagulability in response to having to deal with a supervisor every day is not only ineffective, but the translation of this acute state of readiness into chronic hypercoagulability may result in poor coping and cardiovascular strain [24]. Indeed, this is precisely the situation experienced by trauma-exposed professionals.

The term “stress” is extremely broad. It can refer to external challenges (e.g., work responsibilities), a traumatic physical or psychological event, or an internal experience of strain or negative emotions (e.g., feeling anxious). Furthermore, the experience of stress is necessarily personalized to the individual experiencing it. The same external challenge may result in widely different experiences of stress for different individuals, based upon their discrepant learning histories, personalities, cultural contexts, overall health, and their immediate assessment of the event. Indeed, studies of demographic variables and prothrombotic stress responses have revealed some intriguing findings. Age appears to be positively correlated with prothrombotic stress responses (as measured by the aforementioned D-dimer levels) [25]. Men also appear to have a relatively higher level of stress-induced hypercoagulability as compared to women [26]. Health factors such as hypertension are associated with greater platelet activation and D-dimer increase, suggesting that individuals with elevated rates of cardiovascular disease may experience diminished fibrinolysis activation [27].

What is important for this discussion is that hemostatic alterations can be driven by the experience of stress, with all of the aforementioned variables playing a role. We wish to briefly discuss two of several chronic stressors that are particularly relevant for the functioning of the hemostatic system for trauma-exposed professionals: occupational stress and psychological health.

One’s occupation can directly relate to hemostatic functioning. Some of this comes from the economic consequences of employment. For example, higher levels of fibrinogen are found in unemployed versus employed individuals [28]. This finding dovetails with other research indicating that lower socioeconomic status, including occupation, predicts elevated levels of fibrinogen [29]. However, there is also evidence that these processes are modified by the experience of on-the-job tasks, as opposed to solely economic factors.

An important early demonstration of this finding was conducted by Friedman and Rosenman (1959) [30], who found that accountants experiencing a period of increased workload during the tax season demonstrated decreased coagulation time that was alleviated when the workload was normalized. A number of other studies have found similar results, though it should be noted that there are a great number of complicating factors within these data, and the findings are not universal. Nonetheless, research has repeatedly identified that workplace stress can translate to elevated levels of procoagulants and various prothrombotic markers—an overall state of hypercoagulability (accompanied by decreased fibrinolytic activity). These findings have been observed in situations where there is a discrepancy between workplace expectations and abilities [31], when employees report feelings of exhaustion [32], among civil servants [33], among factory workers [34], and across international boundaries [35]. These findings have also been demonstrated in interpersonal settings, such as those involved in caretaking. Those engaged in caretaking for individuals with Alzheimer’s disease have demonstrated similar hypercoagulability [36], with particularly intriguing research noting that problematic behaviors engaged in by the suffering individual are associated with elevations in a procoagulant index among caretakers [37]. Speaking summarily, there appears to be an important relationship between one’s working environment and hemostatic functioning [38].

Psychological distress and psychiatric symptoms, such as depression and anxiety, are also associated with hypercoagulability [39]. A particularly strong relationship between depression and related thrombophilic states has been observed [40]. Self-reports of depressive symptoms reliably correlate with procoagulant and antifibrinolytic variables [41,42], and depressed individuals demonstrate increased platelet activity [43]. These results have also been reported when following individuals longitudinally [44]. More generally, in a study of healthy young adults, the relation between self-reported distress and increased fibrinogen levels was maintained even after statistically controlling for a number of potential confounds [45].

The literature concerning anxiety and prothrombotic states is more ambiguous. Some studies have found that anxiety is associated with increased fibrinogen levels [31]. In a study of patients with an anxiety disorder (i.e., panic disorder or social phobia), Geiser et al. (2008) calculated summary scores from coagulation and fibrinolysis measures. As compared to controls, patients with anxiety disorders were higher in composite hemostatic scores and a sum score of fibrinolysis. However, these results have not been consistently replicated [46]. A somewhat more consistent finding is that anxiety may relate to impaired fibrinolysis [39].

Those who work with trauma-exposed professionals may be particularly interested in the relation between PTSD and hemostatic functioning. Prothrombotic markers (e.g., soluble tissue factor) may be predictive of the development of PTSD symptoms following a traumatic incident and are similarly relevant for the PTSD symptom cluster of experiential avoidance [47]. Hyperarousal and overall number of PTSD symptoms may also correlate with plasma fibrinogen levels [48]. Severity of PTSD has also demonstrated associations with pro-thrombotic factors, including among civilians with a PTSD diagnosis [49]. However, not all studies have found baseline differences when comparing those with a diagnosis of PTSD against controls [50], with some evidence suggesting that momentary stress may be an important moderating variable [51].

What is clear is that hemostatic functioning can be significantly affected by the experience of occupational strains, be they chronic stressors, physical injury, or psychological pain. Hypercoagulability is a primary indicator of this process and has been utilized in research as a biomarker of an individual’s stress-response. However, comparatively less attention has been devoted to the complementary role of anticoagulants or the response in stress adaptation.

As we previously described, anticoagulants limit the activation of thrombin, an important process to prevent thrombosis. Upon first glance, the relevance of this process may seem limited to the experience of physical injury. Although the mechanism of hypercoagulability likely evolved for functioning in the face of potential injury, increasing evidence suggests it becomes chronically activated in the face of modern workplace stress. Similarly, so too does the body’s system of endogenous anticoagulants have a role to play beyond the specific experience of response to injury.

With the body entering a prothrombotic state, anticoagulants may be released as a natural countermeasure, and this is particularly demonstrated in areas of the blood vessel system with an increased risk for clotting, e.g., where vessels divide or bifurcate, which causes turbulence that increases the risk for clotting [52]. As we have discussed, this is important during stressful encounters in order to limit the risk of stress-induced blood clotting [52]. A 5–10% elevation in antithrombin has been observed following stressful psychological tasks in a laboratory, with return to basal levels seen within 45 min post-stress [53]. In chronic stress, the functioning of anticoagulants has been proposed by some as a counterregulatory measure to balance the prothrombotic effects of chronic stress [54]. In other words, if chronic stress induces hypercoagulability, anticoagulants may be released to attenuate the clotting effects.

In our own research with police officers, we have examined the potential effects of anticoagulant levels. In a previous study [55], we examined the performance of police officers during a simulated crime scenario. The scenario was complex, involving multiple actors, unexpected changes in circumstances, and actors speaking in multiple languages. The participating cadets were evaluated by live observers across a number of domains and blood samples were collected prior to, and immediately following, the simulation.

Because anticoagulants may serve the noted counterregulatory function, they are in some respects an index of physiological arousal (i.e., levels elevate in response to the prothrombotic state brought on by the experience of stress). In our study with police cadets, elevations in the anticoagulant antithrombin predicted higher Total Performance Ratings (a summary score of the cadet’s overall performance across multiple skill domains). Antithrombin was also a significant predictor of performance in Tactical Skills and Nonverbal Communication. Conversely, antithrombin levels were negatively correlated with Verbal Performance, a finding that we attributed to the differential effects of physiological arousal on performance of tasks with high cognitive demand.

These positive effects of antithrombin are important to note. If the assertion that anticoagulants serve as a counterregulatory measure to the prothrombotic state of stress is true, then anticoagulants may represent more than a proxy for the experience of stress and serve as a specific indicator of the body’s healthy response to stress. As such, when measuring the health of professionals, be it in response to an acute event or to ongoing workplace strains, the measurement of anticoagulants may provide an index of the extent to which the body is able to adaptively respond to some of the more commonly assessed hemostatic markers.

## 6. Measurement Advice

As evidenced from the current discussion, the hemostatic system is complex. However, at a higher level, there are three distinct components: the platelets; the coagulatory systems; and the anticoagulatory systems.

The simplest test is to measure bleeding time, which is a reflection of the clotting system’s efficiency. The shorter the bleeding time, the more active the clotting system is. The two most commonly used measures of clotting time are prothrombin time (PT) and activated partial thromboplastin time (aPTT). These tests are widely available at most clinical pathology laboratories. These measures are also used to make sure that persons treated by blood thinners (e.g., after having suffered a thrombolytic stroke) receive the right amount of anticoagulant medication.

The concentration of platelets in the blood is part of the complete blood count panel often reported by primary care doctors as part of the annual physical. The normal range, partly dependent on lab reference values, is between 140 and 400 × 10^3^/uL. In addition to measuring the number of platelets in the blood, there are methods to determine how well they function.

Measurement of various components of the coagulatory and the anticoagulatory secondary homeostatic systems is also well-established and is performed in most clinical pathology laboratories. As discussed above, the secondary homeostatic system can be likened to a cascade. Dependent on clinical concerns and specific research interests, one has to select which of several components in the coagulatory and the anticoagulatory systems one wants to measure.

However, in the case of capturing the overall function of the clotting system and changes over time due to stress, measuring the clotting time is a simple, straightforward, and rather inexpensive method. Measuring other components and systems discussed above is only motivated with the need to understand damages to specific components of the clotting systems.

## 7. Conclusions

As researchers and clinicians consider new methods and variables to supplement self-report measures of stress and health among trauma-exposed professionals, we hope that anticoagulants will begin to receive increased attention. As a core biological process, anticoagulants may serve as an important biomarker of response to stress, both acutely and chronically. Furthermore, the methods to assess these processes can be relatively straightforward and inexpensive, particularly for those already accustomed to blood sample collections. Although anticoagulation and its associated mechanisms may initially appear somewhat alien, as the preceding discussion has hopefully illustrated, it is a conceptually and practically accessible subject for research and clinical attention.

## Data Availability

Not applicable.
